# Hybrid Nanofluids as Renewable and Sustainable Colloidal Suspensions for Potential Photovoltaic/Thermal and Solar Energy Applications

**DOI:** 10.3389/fchem.2021.737033

**Published:** 2021-09-27

**Authors:** Tahir Rasheed, Tariq Hussain, Muhammad Tuoqeer Anwar, Jazib Ali, Komal Rizwan, Muhammad Bilal, Fwzah H. Alshammari, Norah Alwadai, Amani Saleh Almuslem

**Affiliations:** ^1^ Interdisciplinary Research Center for Advanced Materials, King Fahd University of Petroleum and Minerals (KFUPM), Dhahran, Saudi Arabia; ^2^ Department of Mathematics, Faculty of Basic Sciences, University of Wah, Wah Cantt, Pakistan; ^3^ Department of Mechanical Engineering, COMSATS University Islamabad, Sahiwal Campus, Sahiwal, Pakistan; ^4^ Electronic Engineering Department, University of Rome Tor Vergata, Rome, Italy; ^5^ Department of Chemistry, University of Sahiwal, Sahiwal, Pakistan; ^6^ School of Life Science and Food Engineering, Huaiyin Institute of Technology, Huaian, China; ^7^ Department of Physics, University of Hafr Al Batin (UHB), Hafr Al Batin, Saudi Arabia; ^8^ Department of Physics, College of Sciences, Princess Nourah Bint Abdulrahman University (PNU), Riyadh, Saudi Arabia; ^9^ Department of Physics, College of Science, King Faisal University, Al-Ahsa, Saudi Arabia

**Keywords:** hybrid nanofluids, colloidal solutions, nanoparticles, solar cells, photovoltaic thermal systems

## Abstract

The comparative utilization of solar thermal or photovoltaic systems has significantly increased to fulfill the requirement of electricity and heat since few decades. These hybrid systems produce both thermal and electrical energy simultaneously. In recent times, increasing interest is being redirected by researchers in exploiting variety of nanoparticles mixed with miscellaneous base fluids (hybrid nanofluid) for these hybrid systems. This new class of colloidal suspensions has many fascinating advantages as compared to conventional types of nanofluids because of their modified and superior rheological and thermophysical properties which makes them appealing for solar energy devices. Here, we have attempted to deliver an extensive overview of the synthetic methodologies of hybrid nanofluids and their potential in PV/T and solar thermal energy systems. A detailed comparison between conventional types of nanofluids and hybrid nanofluids has been carried out to present in-depth understanding of the advantages of the hybrid nanofluids. The documented reports reveal that enhanced thermal properties of hybrid nanofluids promise the increased performance of solar thermal PV/T systems. Additionally, the unique properties such as nanoparticles concentration and type of base fluid, etc. greatly influence the behavior of hybrid nanofluidic systems. Finally, the outlook, suggestions, and challenges for future research directions are discussed.

## Introduction

The widespread growth of industrial and world’s population results in the global energy crisis. The significant developments of society may result in a humanitarian disaster. The consumption of conventional petroleum fuels is rapidly increasing, whereas, its accessibility is constantly diminishing. Currently, bio-renewable energy produced from sustainable means, such as wind, geothermal and solar energies is an attractive substitute for the fossil fuels (Fudholi et al., 2019). Among these resources, solar energy is important source for renewable energy in the production of heat and electricity. Several solar-assisted thermal systems are being employed to producing electricity, water heating, air-conditioning, and desalination. In designing these solar energy systems (SESs), the most significant factor is to improve the heat transfer capability for increased performance. The fluids having increased thermophysical character can be better candidates for the aforesaid purpose. Among these fluids, the nanofluids are the best fluids to achieve the enhanced heat transfer properties of the fluid. The term nanofluids is referred to the fluids in which nanoparticles (NPs) having particles size in the range of 1–100 nm are suspended in a base fluid (water, oil or organic liquids) (Amalraj and Michael, 2019). At present, a plethora of reports have already been reported in which researchers have used the nanofluids in absorption refrigeration systems, solar stills, solar cells, water heaters, solar collectors, and solar cooling systems as a blend of various solar devices owing to their exceptional properties than the conventional fluids. The advantages of the nanofluids in The SESs are presented as follows:

1. The NPs having smaller size and greater surface area can greatly affect the heat capacity and absorption of nanofluids for solar energy devices.
2. In comparison to base fluids, nanofluids have better optical properties. In both infrared and solar spectrum, they exhibit low emittance and high absorption.
3. The presence of NPs in base fluid increases its thermal conductivity. Therefore, nanofluids possess enhanced thermal conductivities relative to the base fluids.
4. The high stability and high absorption coefficients make nanofluids an excellent absorption medium under wide range of temperature gradients.
5. The extreme small size of NPs in nanofluids results avoidance of obstruction, sedimentation, fouling of pumps and pipes. This property makes nanofluids an ideal candidate for solar applications.
6. The heat transfer areas are decreased by nanofluids in thermal devices, this results in the cost effectiveness of the SESs.
7. Generally, nanofluids exhibit better convective heat transfer coefficient and high density with a lower specific heat of NPs which ultimately increases the efficiency of thermal devices.

**Graphical Abstract:**
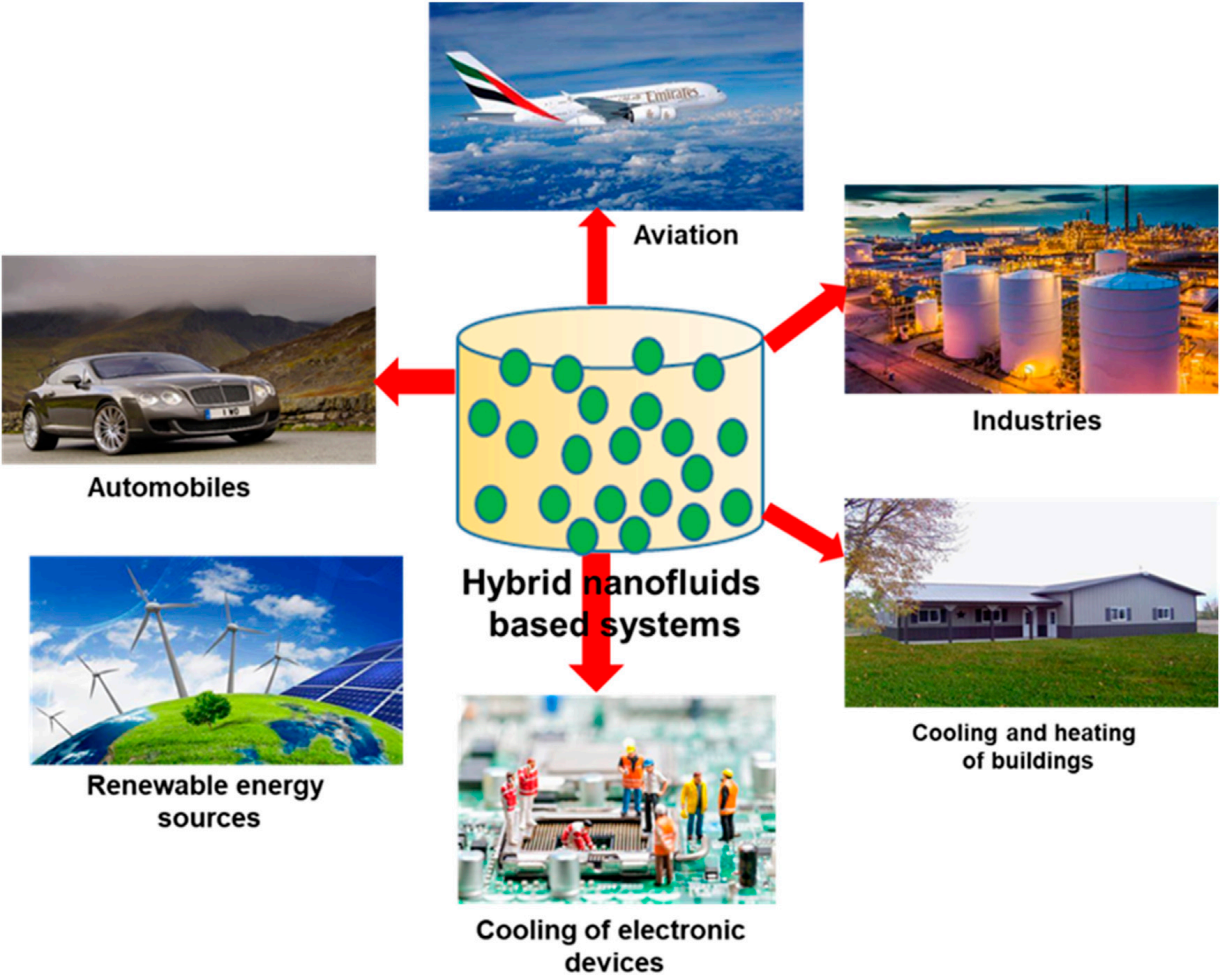


The effect of the size and volume fraction of NPs in heat transfer have been extensively inspected by various researchers. For instance (Xie et al., 2010; Moraveji et al., 2011; Wongcharee and Eiamsa-Ard, 2011), investigated on CuO-H_2_O based nanofluids. They used a laminar system by taking the three volume fractions (0.3, 0.5, and 0.7%) of the nanofluidic system. The findings indicate that the Nusselt numbers are improved by increasing the concentration of nanofluids. In another approach, Santra and co-workers (Santra et al., 2009) used the similar system (CuO-H_2_O) for solid volume fraction (0.00 and 0.05) and a wide range of Reynolds numbers (up to 1,500). The group considered the fluid in two phases such as Newtonian and non-Newtonian. The observations revealed that the heat transfer greatly enhanced by raising the volume fraction. Arani and Amani (Abbasian Arani and Amani, 2012) investigated the TiO_2_-water based nanofluid system by carrying out the experimental investigations for Reynolds numbers and volume fraction. The results revealed an increased heat transfer with increased concentration of volume fractions. Further, they found that at higher values of Reynolds numbers, extra power is required to astound the drop of pressure of nanofluid. Therefore, they concluded that the lower values of Reynold numbers were more beneficial as compared to higher values for using the nanofluids. Fotukian and Nasr-Esfahany (Fotukian and Nasr Esfahany, 2010) examined the γ-Al_2_O_3_-H_2_O nanofluid for heat transfer features. The heat transfer was not affected by increasing the solid fractions while the addition of NPs enhanced the thermal conductivity. In another approach, Sebdani and co-workers (Lelea, 2011) documented Al_2_O_3_-water system in a square crater for mixed convection at fixed Rayleigh numbers. The outcomes revealed the heat transfer drop for small Reynolds number (Re¼1) whereas fraction of volume was >0.05. On the other hand, at higher values of Reynolds number (10–100) the increased concentration of NPs increased the heat transfer. Correspondingly, for a persistent Reynolds number, the influence of addition of NPs on heat transfer was associated with Rayleigh number. The addition reveals that the heat transfer increases up to Ra_¼_10^3^ whereas the decrease in heat transfer was noticed for Ra_¼_10^4^ and Ra_¼_10^5^ with continuous addition of more NPs (Fotukian and Nasr Esfahany, 2010). The influence of the diameter of NPs on the thermal conductance of nanofluids was investigated in a number of reports. Lelea (Lelea, 2011) investigated the numerical modeling at constant Reynolds numbers using a heat sink of microchemical size. The investigation revealed that the transfer of heat reduced by increasing the diameter of Al_2_O_3_ NPs in a base fluid. In another investigation, Teng and others (Teng et al., 2010) examined the impact of temperature and size of NPs using Al_2_O_3_-water based nanofluid system. They confirmed improved thermal conductivity in NPs with a reduced diameter. The most fascinating feature of this investigation was the increased heat transfer at higher temperatures. On the contrary, Beck et al. (2009) found the decrease in thermal conductance by reducing the diameter of NPs in ethylene glycol-Al_2_O_3_ and water-Al_2_O_3_ systems. The similar outcomes were found for H_2_O-Au based nanofluid (Shalkevich et al., 2010). Another important use of nanofluids is as coolants for the electronic devices. In recent times, they prove to be best candidates in improvement of thermal conductivity in heat sinks (Bahiraei et al., 2019a; Bahiraei et al., 2019b; Farzanehnia et al., 2019). In a small channel heat sink, Ijam and Saidur (Ijam and Saidur, 2012) carried out the investigations for TiO_2_-water and SiC-water based nanofluids as coolant. The findings revealed an enhanced thermal conductivity as compared to base fluid. An electronic heat sink was used (Selvakumar and Suresh, 2012) for CuO-H_2_O system. The group observed similar results as discussed above. Similarly, Hung and Yan (Hung and Yan, 2012) reported the increased thermal performance by the use of nanofluids. They used the double layered microchannel heat sink for Al_2_O_3_- H_2_O system. Due to the speedy growth in world-wide population, the demand of the various energy sources is not sufficient. As the sources of fossil energy are being restricted to the specific area of life, therefore, more and more interest is developed in solar energy to meet the energy requirement as a suitable and environmentally friendlier substitute. In solar energy systems, the low system efficiency is major area to focus on; henceforth, collecting solar radiation with great efficiency is the paramount issue. This deficiency has greatly been filled by nanotechnology by using the nanofluid as some important candidate to increase the efficacy in solar systems.

Herein, we have made an attempt to review the data of the reported investigations on nanofluid in solar systems applications. A number of review articles have been published till now but most of them discuss the properties of nanofluids and their applicability in some engineering fields (Suleimanov et al., 2011; Razali et al., 2019; Alarifi et al., 2019; Asadi et al., 2019; Nunes et al., 2019; Sahu and Sarkar, 2019; Gholamian et al., 2020). This review explicitly presents the current research trends on the exploitation of nanofluids in SESs. The first part of the review focuses on the synthetic approaches used for the preparation of nanofluids. In second part, the general characteristics of the nanofluidic systems have been summarized. Third part of the review deals with the applications of nanofluids in SESs such as solar cells, solar collectors, and solar stills. The last part of the review discusses the purposed future work with current challenges and limitations of hybrid nanofluids in SESs.

## Classification, Fabrication, and Characteristics of Hybrid Materials

On the basis of matrix, the hybrid materials are classified into three categories.

(1) Metal-based nanocomposites.

(2) Ceramic-based nanocomposites.

(3) Polymer-based nanocomposites.

In this section, we summarize the synthetic approaches used for the preparation of nanofluids and their dynamic characteristics as synthetic methodology plays significant role in determining the characteristics and features of hybrid nanofluid. These hybrid nanofluids can be synthesized by two strategies i.e. single step and two step method (Ranga Babu et al., 2017).

### One-Step Strategy

In single step approach, all the contemporary actions are carried out in one step. The nanofluids prepared from this method generally have high quality and physically stable in relation to physical specifications and thermal characteristic. It is a controlled methodology to produce nanofluids, which contains nanosized NPs dispersed in base fluid. There exist a number of one-step methods, including liquid chemical method and physical vapor deposition (PVD). The main one-step process comprised direct evaporation in one step that was reported (Akoh et al., 1978). Later on, this process was further modified (Eastman et al., 1996) by Eastman et al. He carried out the experiment in which he condensed the copper vapors to copper nanoparticles directly by creating contact with ethylene glycol (EG) as a base liquid having low vapor pressure. Likewise, Aberoumand and Jafarimoghaddam (Aberoumand and Jafarimoghaddam, 2018), used the single step method for the preparation of WO_3_-Ag as NPs and transformer Oil as base fluid. By using electrical explosion wire methodology, 99.9% of pure WO_3_ NPs were dispersed in transformer oil to procedure WO_3-_transformer oil nanofluid. The silver NPs were assorted by the passage of high voltage over evaporative tinny cable of Ag that dispersed in WO_3-_transformer oil as base fluid followed by electrical explosion which resulted in the formation of WO_3_-Ag-transformer oil hybrid nanofluid. At 100 °C, the heat conductivity of the hybrid nanofluid increased by 41% at 4 wt%. Suresh et al. (2011) utilized the combination Cu(NO_3_)_2_-3H_2_O and Al(NO_3_)_3_-9H_2_O for the preparation of powdered Al_2_O_3_-Cu hybrid NPs. The as prepared nanocomposite powder was further heated at a high temperature of 900°C for 1 h to finally obtain CuO-Al_2_O_3_ powder mixture.

### Two-Step Strategy

The two-step strategy is widely adopted to prepare hybrid nanofluids. In two step methodology, the powder of nanoparticles is suspended into base fluids. The nanoparticles used in this method are commercially available or prepared separately by different chemical, mechanical or physical methods such as milling, grinding, sol-gel (Shafi et al., 2019), etc. After that, these nanoparticles are suspended in suitable base fluids by ultra-sonication, magnetic stirring, homogenizing and high-speed mixing.

### Dispersion Techniques

**FIGURE 1:**
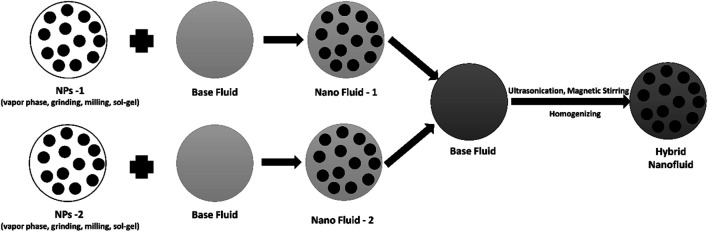
Schematic illustration of two step synthetic approach.

The nanofluids stability is greatly impacted by NPs dispersion in base fluids. The well dispersed and homogenized suspension lead to the greater stability to the nanofluidic system. Several techniques comprising ultrasonic bath, ultrasonic disrupter, high-pressure homogenizer and stirrer are frequently employed for well dispersion of NPs. In recent times, Hwang et al. (Lu et al., 2008), used the amended magnetron sputtering system. They mixed the sputtered NPs directly. Further, novel approaches consist of preparation of a specific kind of nanofluids using surface functionalization, aqueous organic processing, and acid treatment. These methods were applied for enhancing the NPs compatibility with base fluids. Figure 1 illustrate the synthetic methodology of two step approach.

### Effect of Surfactants on the Behavior of Hybrid Nanofluids

**FIGURE 2:**
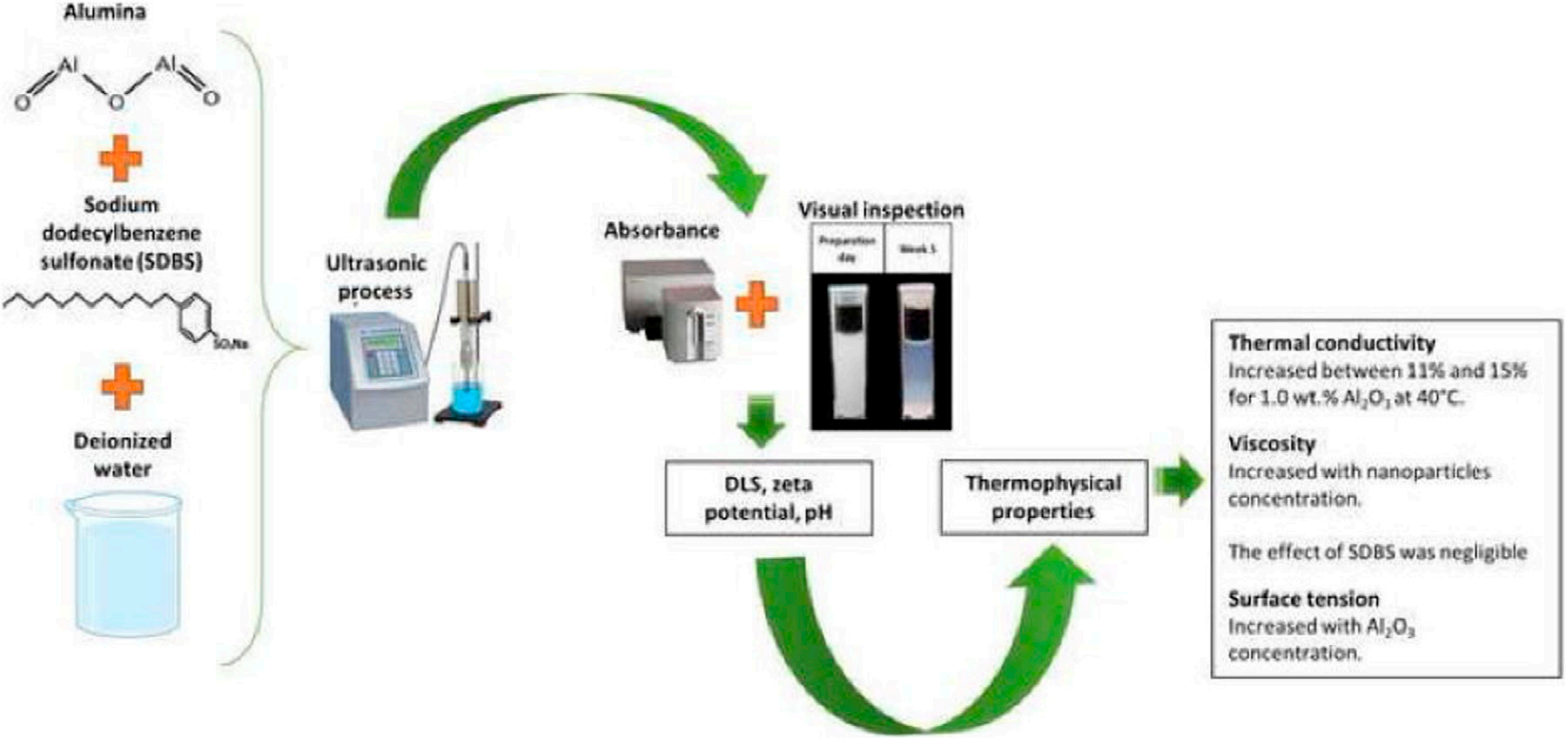
Schematic Illustration of water-Al_2_O_3_ based nanofluids using SDBS as dispersant agent (Gallego et al., 2020).

Substances that greatly decrease the surface tension of a liquid are surfactants or surface-active agents, resulting in the distribution of one phase to another. This concept is not limited to solid suspensions of liquids and can include liquid-liquid systems as well. As the architecture of surfactants consists of negative and positive part, so normally these agents accumulate on the interfacial layer. The preferential orientation of these molecules is that the soluble portion binds to the liquid and the insoluble portion bonds to the solid surface. Therefore, it can be concluded that the addition of dispersants or surfactants is economical and easy way to enhance the nanofluids stability. The nanofluids without surfactant or dispersant exhibit higher sedimentation as compared to the nanofluids having surfactants. The mechanism behind this stable suspension involves the creation of some bonds or physical interactions among nanoparticles and base fluid. The presence of surfactants into base liquid covers the nanoparticle surface which results in the reduction of aggregation of nanoparticles (Lu et al., 2008; Saeedinia et al., 2012). Despite the numerous studies on single nanofluid containing surfactants, only a few studies on the impact of surfactants on hybrid nanofluids have been conducted so far. More precisely, the effect on the thermophysical properties, rheology and stability of hybrid nanofluids by adding the surfactant and its concentration has not been studied in detail. Review of existing literature suggests that many experiments on the thermophysical properties of hybrid nanofluids have been carried out so far to evaluate the concentration and materials of various surfactants. Some of the documented reports reveal that the addition of surfactants shows negative impact on the thermophysical properties of hybrid nanofluids. The report presented by Gallego and co-workers reveals that the addition of SDBS (0.32 wt%) to a hybrid nanofluidic system containing Al_2_O_3_ shows increased temporal stability, while the thermal conductivity and surface tension reduced greatly (Gallego et al., 2020) (Figure 2). In another approach, the addition of PVP to a TiO_2_/W nanofluid improved the dispersibility and the viscosity was also increased which effects the thermal conductivity. On the other hand, some of the reports documented that the addition of surfactant can have a positive impact on the thermophysical properties of the nanofluidic system. For example, different weight concentrations of surfactant were used by Arasu and co-workers to examine the thermophysical properties and stability of the TiO_2_-Ag/W based nanofluidic system. They observed an enhancement of thermal conductivity up to 29.6 and 2.1% by using 0.1 wt% of SDS and SDBS surfactants, respectively (Arasu et al., 2019). Similarly, an increase of thermal conductivity by 9.8% was observed by Leong and co-workers by the addition of PVP to Cu-TiO_2_ (0.8 wt%) system. The literature survey shows that the inconsistencies in the nature and concentration of the surfactant can be the source of the thermophysical properties of nanofluids. More research is needed in the thermophysical characteristics of hybrid nanofluids. It is required to deeply investigate the impact of the complex interaction between nanoparticles and surfactant molecules on the stability and thermophysical properties of hybrid nanofluids in order to address the aforementioned shortcomings.

## Advantages, Disadvantages and Requirements of Different Base Fluids

Low viscosity and greater thermal conductivity of a base fluid guaranty the better results for heat transfer applications. These fluids (heat transfer) find substantial uses in a number of industrial zones such as electric transformers, cooling of engines, spray cooling, power sector, machining processes, etc. The dynamic and submissive approaches are being used by the researchers to elevate the heat transferring capacity of a base fluid since last few decades (Gupta et al., 2009). On the other hand, these approaches have touched their bottleneck. Currently, the scientists are working on the methods to improve the thermophysical attributes of a base fluid by engaging NPs in it. Stability is another important factor (primary factor) to obtain better heat transfer effects while nanofluids are investigated. The electro-kinetic character of a base fluid directly relates with stability. Similarly, by controlling the pH, the stability of a nanofluid can also be increased. Water is better base fluid as compared to other fluids. Wen et al. (Gupta et al., 2009) revealed that stability of CNTs was fairly good when treated with water. Comparatively, the nanofluids based on deionized (DI) water have significant improvement in heat transfer than EG-based nanofluids (Sanchez et al., 2005; Jyothirmayee Aravind and Ramaprabhu, 2012; Theres Baby and Sundara, 2013). The nanofluid based on biological system was presented by Sarfaraz and co-workers (Sarafraz and Hormozi, 2015). The group revealed that the 1% vol. of biological base fluid had greater thermal conductivity compared to other base fluids. Similarly, the investigations carried out by Sundar’s group (Syam Sundar et al., 2017) revealed 19.14% increase in thermal conductivity of water-based nanofluidic system compared to EG-based system at 0.2 vol%. Another innovative class of nanofluids is known as hybrid nanofluids. These types of fluidic systems are synthesized by amalgamating two or more than two different types of NPs in one base fluid or a hybrid composite. These systems are still under research or in developing phase according to industrial viewpoint. The performance of such systems is expected to be very good in a variety of applications (Moghadassi et al., 2015).

### Water-Based Hybrid Nanofluids

Water as a base fluid has been reported by a number of investigators for the development of hybrid nanofluids. Enhanced thermal properties have been reported by all the investigators when compared with the systems having single NPs-based nanofluids. Here, some of the reports based on these studies are presented. Abbasi and co-workers (Abbasi et al., 2016) utilized the water as base fluid and prepared MWCNTs-TiO_2_ NPs with 5–15 nm in length and size in the range of 40–60 nm. The group carried out the hydrolysis of TiCl_4_ to prepare the TiO_2_ NPs. High speed stirring and ultrasonication was utilized for the acid treatment of MWCNTs and TiO_2_ NPs. Furthermore, the TiCl_4_ was added dropwise to fabricate the hybrid NPs of MWCNT-TiO_2_. The hydrolysis of TiCl_4_ was carried out by the addition of HCl to the water. Similarly, the CuO-Cu nanofluid was prepared by Balla et al. (2013) using two step strategy. The group used various concentrations (1, 0.8, 0.6, 0.4 and 0.2%) of base fluids. The mixing of the suspension was completed by ultrasonication. The average size of the NPs was found to be around 50 nm. No stabilizer and additive were used during the synthetic process. CNT-TiO_2_ based hybrid nanofluid was reported by Megatif et al. (2016). The group utilized the modified hydrolysis approach. The functionalization of CNTs was carried out by using H_2_SO_4_ and HNO_3_ solution in a volume-to-volume ratio of 3:1. The well dispersed framework of CNTs was obtained at 70°C for 3 h. These treated CNTs were dispersed in water as base fluid using ultrasonication for a period of 30 min. The solution pH was maintained at 1.5. Furthermore, the 2-propanol and EG were added to the mixture upon continuous stirring followed by the addition of Ti(OBu)_4_. Finally, the dark colored precipitates were obtained and separated through vacuum filtration. The CNT-TiO_2_ composite was obtained after calcination. Madhesh and Kalaiselvam (Madhesh and Kalaiselvam, 2015) synthesized the hybrid nanocomposite based on copper-TiO_2_. The experiment was carried out by the dispersion of titania in water and finally adding copper acetate through vigorous stirring in the presence of ascorbic acid and sodium borohydride, which acted as reductants. The subsequent solution was kept stagnant for 2 h to acquire the colloids of hybrid nanocomposite. The obtained mixture was washed thoroughly, filtered and vacuum dried. Finally, the material obtained was re-dispersed in water to obtain hybrid nanofluid. Chemical reduction and *in situ* approach were used by Sundar et al. (2016). The group prepared Nano diamond (ND)-Fe_3_O_4_ nanofluids. The impurities from nano diamond were removed by immersing the soot of ND in concentrated sulphuric acid followed by magnetic stirring and continuous washing trailed by drying for 72 h. Finally, the ferric chloride salt was added slowly by stirring for 2 h. After completion of reaction, ND-Fe_3_O_4_ was washed extensively with water to eliminate the extra amount of reagents left behind.

### Oil-Based Hybrid Nanofluids

**FIGURE 3:**
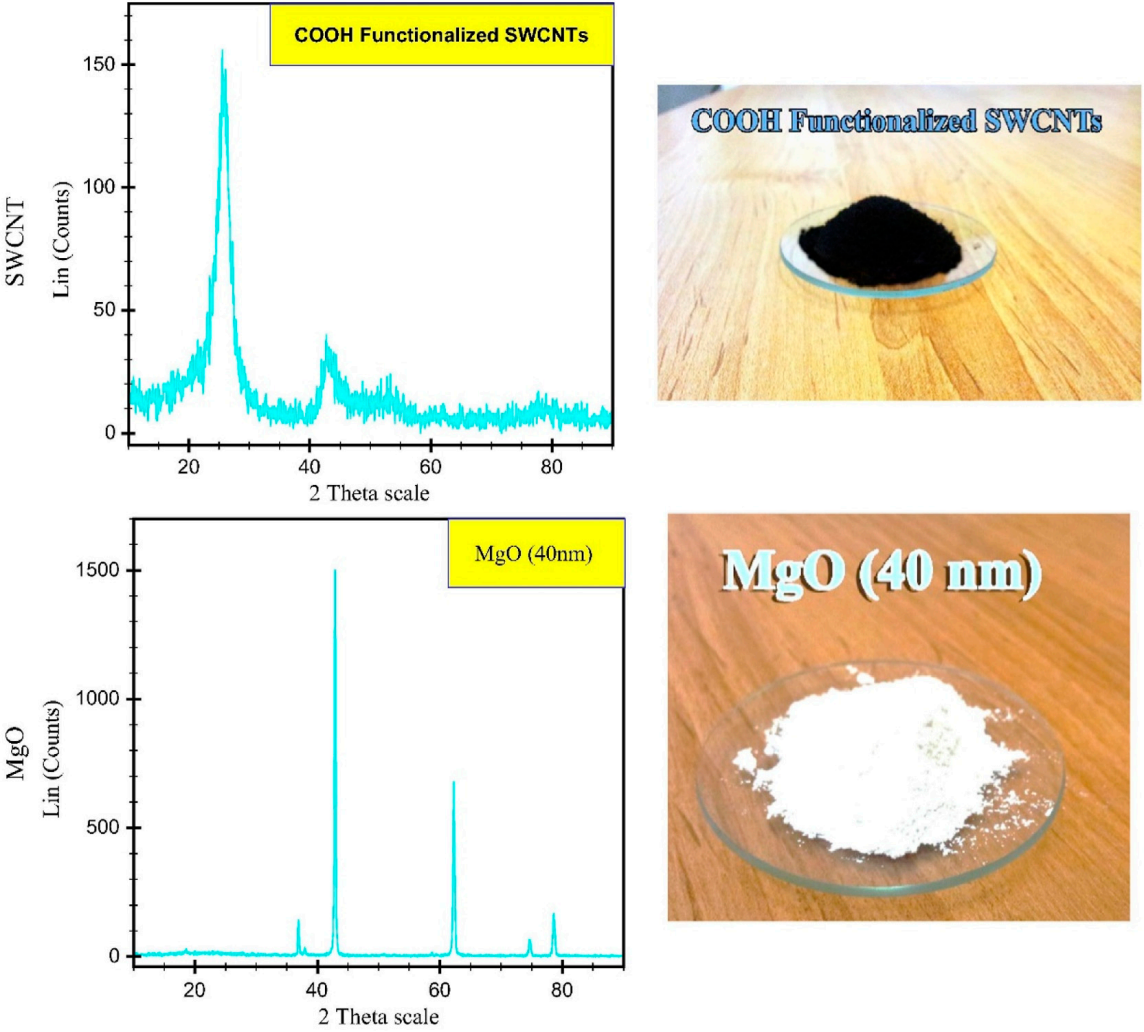
XRD analysis of prepared nano powders (Hemmat Esfe et al., 2017).

Oil as a base fluid has been used by a variety of researchers and it has been claimed that the thermal properties of hybrid nanofluids are superior to nanofluids with single NPs or base fluids. Here we will discuss few examples of such reports. The *in-situ* approach was used by Kumar et al. (2016) for the preparation of Cu-Zn (50:50) hybrid nanofluids. Spherical shaped particles (average size 25 nm) were observed by using SEM and TEM techniques. After that the certain amount of nano-powder was dispersed in vegetable oil by ultrasonication for 2 h obtaining the hybrid nanofluid. Hemmat Esfe et al. (2017) used two-step approach for preparing SWCNTs-MgO hybrid nanofluids (Figure 3). The calculated masses of all the materials were dispersed in engine oil. The stability of the suspension was carried out by ultra-sonication. The nanofluids witnessed negligible sedimentation and outstanding stability.

### EG-Based Hybrid Nanofluids

The nano diamond and nickel-based hybrid nanofluid were prepared by Sundar et al. (2014). The group utilized the *in-situ* approach. Carboxylated nano-diamond NPs were dispersed in EG, followed by the addition of nickel chloride. The NaBH_4_ was applied as reducing agent and the reaction happened at a temperature of 1,400°C. The homogenous mixing of the suspension was attained by using nano-spheres followed by ultrasonication for 1 h. Furthermore, the magnetic nanofluids based on ND-Ni nanocomposite were prepared by using different concentrations of water and EG (water, EG, 20:80%, 40:60%, 60:40%). Similarly, by using EG, Paul and co-workers (Paul et al., 2011) prepared Al-Zn NPs with the help of mechanical alloying. The uniformity of powder was obtained by using milling. The addition of NPs powder in certain amount of base fluid was carried out by using ultrasonication followed by magnetic stirring. In another work, activated carbon and graphene-based hybrid nanofluids (ACG/EG) were synthesized by Yarmand et al. (2016) by using EG as base fluid. The graphene flakes were used for the preparation of graphene oxide using standard Hummers method (Chen et al., 2013). At a temperature of 500°C, Pyrolysis was carried out trailed by formation of carbon products through ball milling. Subsequently, GO having 3 wt% contents of carbon was dispersed in distilled water by the use of sonication for 30 min. Carbon and GO were variegated with potassium hydroxide solutions and were stirred for 2 h. The samples were heated up to 880°C after careful mixing followed by successive washing for eliminating impurities to yield ACG nanocomposite. Similarly, the GO/Co_3_O_4-_based hybrid nanofluids were synthesized by Sundar et al. (Che Sidik et al., 2017). The GO/Co_3_O_4_ hybrid NPs were prepared by using chemical reaction and *in situ* approach. The Functionalized GO nanosheets were dispersed in 100 ml of distilled water by sonicating them in sonication bath. After that, calculated amount of CoCl_2_.6H_2_O solution was mixed in another flask or beaker with continuous stirring till Co^2+^ ions got meticulously dispersed in the solution. Sodium borohydride was added into the solution of GO/Co^2+^ to remove the chloride, sodium and boron. Precipitates were thoroughly washed, filtered and dried. The detailed investigations carried by various researchers by using various base fluids and nanoparticles are presented in Table 1.

**TABLE 1:** Summary of hybrid nanofluids synthesized using different nanoparticles.

Base fluid	Surfactant used	Preparation method	Dispersion mode	Nanoparticles	References
Water	Without any surfactant	Two step method	Ultrasonication	Graphene oxide	Yang et al. (2019)
Vegetable oil	Without any surfactant	*In situ*	Ultrasonication	Cu-Zn	Dhinesh Kumar and Valan Arasu (2018)
Distilled water	Without any surfactant	Two-step method	—	Ag and TiO_2_	Batmunkh et al. (2014)
Ethylene glycol	Without any surfactant	Two step method	—	Fe_3_O_4_-Ag	Afrand et al. (2016)
Ethylene glycol	Without any surfactant	Mechanical alloying	Ultrasonication	Al-Zn	Paul et al. (2011)
Distilled water	Nanospheres	*In situ* growth and chemical coprecipitation	Ultrasonication	MWCNT and Fe_3_O_4_	Syam Sundar et al. (2016)
Engine oil (SAE40)	Without any surfactant	Two step method	—	SiO_2_-MWCNT	Hemmat Esfe et al. (2016)
Water	Without any surfactant	Two step	Ultrasonication	ZnO-Ag	Esfahani et al. (2018)
Distilled water	SDBS	Calcinated	Ultrasonication	CNT-TiO_2_	Megatif et al. (2016)
Deionized water	Without any surfactant	—	Ultrasonication	Ag/MWCNTS	Farbod and Ahangarpour (2016)
Water	—	Two-step method	—	Nanodiamond-Fe_3_O_4_	Syam Sundar et al. (2016)
Ethylene glycol	Without any surfactant	Simple heat treatment rout	Ultrasonication bath	Activated carbon graphene composite (ACG)	Yarmand et al. (2016)
Water, Ethylene glycol, Water ethylene glycol mixture	Without any surfactant	*In situ* and chemical co-precipitation method	Ultrasonication bath	Graphene oxide (GO)/Cobalt oxide (Co_3_O_4_)	Syam Sundar et al. (2017)
Distilled water	Without any surfactant	Two step method	Ultrasonic vibrator	γ-Al_2_O_3_	Karimzadehkhouei et al. (2019)
Distilled water	No surfactant	Hydrolysis	Ultrasonic bath	MWCNT-TiO_2_	Abbasi et al. (2016)

## Characteristics of Hybrid Nanofluids

The characteristic of hybrid nanofluids have been investigated by a number of researchers and almost all the investigators have documented promising results. These documented reports have proven that the hybrid nanofluids are among the strongest candidates for solar systems which necessitate good rheological, optical, and thermal properties of working fluid. This section elaborates morphological, thermal, optical, and rheological attributes of hybrid nanofluids as documented in the current literature.

### Thermal Characteristics

The good thermal properties of a fluid are of utmost importance for application in SESs systems such as solar collectors or PV/T systems. Various investigations addressing the assessment of heat transfer or thermal conductivity of hybrid nanofluids are presented. Hemmat Esfe and co-workers (Hemmat Esfe et al., 2015) investigated the thermal conductivity as a function of temperature and concentration of nanofluids by using SiO_2_-MWCNT/EG system. The group carried out the investigation both numerically and experimentally. The variation of concentration and temperature was in the range of 0.05–1.95 vol% and 30–50°C respectively. An improvement of 22.2% in thermal conductivity was observed at 1.95 vol% and 50°C collectively. Increased concentration of nanofluid enhanced the amount of NPs, which triggered prodigious improvement of thermal conductance. Escalation of hybrid nanofluid temperature improved Brownian motion of suspended NPs and consequently improved collisions happened, which ultimately increased the thermal conductivity of the hybrid nanofluid.

### Optical Properties of Nanofluids

The inclusion of NPs enhances the optical features of the base fluid used in the formation of nanofluids. Various factors, such as shape, volume, size, and concentration play a pivotal contribution to the optical properties of the nanofluids. This feature makes them susceptible to be tailored for an array of applications (Lomascolo et al., 2015). For example, the effect of nanoparticle volume was investigated by the Taylor and co-workers. The group found that the high fraction of volume can absorb the incoming light from the solar radiations in thin layered material. This absorbance of energy resulted in the loss of the thermal energy of the system to the environment. On the other hand, if the particles are laded to a smaller extent, the absorption of energy is not possible, therefore, the nanofluids will be unable to absorb the solar energy (Taylor et al., 2011). Hence, we can say that the nanofluids’ optical properties play vital role in the development of an effective and efficient solar thermal collector. Extinction co-efficient is another important optical property for the nanofluids. Like absorbance and scattering, various factors also effect the extinction co-efficient such as, nature and size of the NPs, dielectric constant of the fluid, concentration of NPs, as well as temperature of the system. It is worth mentioning that the extinction coefficient is the sum of scattering and absorption coefficients. Hossain’s group (Sajid Hossain et al., 2015) reported that the absorption is referred to the loss of the incident light when passed through the medium. For nanofluids, the absorption of the NPs and base fluid is considered to compute the total absorption of the system. The group also found that this might not be taken as a final value as the absorption of both moieties might be unusual in base fluid. Furthermore, adding nanoparticles to the base liquid offers added hurdles to the incident light that force the light to diverge its commencing path. The outcome of scattering is insignificant if the diameter of NPs is less than 10 nm. They have more pronounced effect if the size of particle is greater (Noguez, 2005). When the volume fraction of nanoparticle is less than 0.6%, the scattering effect can also be neglected. The well dispersed NPs cause very small scattering. In another study, Wu and co-workers (Wu et al., 2015) presented that a vital role can be played by surface Plasmon resonance (SPR). This can affect the extinction coefficient to a greater extent during the scattering and absorption processes. The SPR originates from the oscillation of electron exposed to incident light. The group also found that the sharp absorption and scattering peaks were produced because of restricted Plasmon surface resonance in nanostructured or metallic NPs. Similarly, the robust electromagnetic near-field increment is formed when the nanoparticles have smaller size than the incident wavelength.

### Rheological Properties

Rheological properties mainly consist of viscosity and density. As far as viscosity is concerned, it affects the convective heat transfer, pressure drop and pumping power. This section focuses on the different factors which lead to viscosity changes. Soltani et al. analyzed the effects of temperature and concentration of particles on the viscous properties of MgO-MWCNTs dissolved in ethylene glycol. It was observed that the prepared hybrid nanofluid exhibited Newtonian behavior under temperature (30–60°C) and concentration changes (0–1%). It was also noted that dynamic viscosity increased with the increase in concentration of particles while it decreased with rise in temperature (Soltani and Akbari, 2016).

Density of the hybrid nanofluid is also of prime importance as it directly influences frictional factor, Reynolds number, pumping power and other heat-related properties. Ho and co-workers investigated hybrid nanofluid consisting of MEPCM and Al_2_O_3_ and concluded that the density was estimated well with the help of mixing model. It was interesting to note that the density increased with the increase in nanoparticles and decreased to some extent with the increase in MEPCM (Ho et al., 2010).

### Morphological Properties

These properties deal with shape, structure and size of material. The shape of the nanoparticles is analyzed through spectroscopic techniques and electron microscopes. Several studies have been conducted to know the effect of shape on nanofluid operation. It has been observed that cylindrical particles tend to have greater values of thermal conductivity. For instance, CNTs exhibit thermal conductivities in the range of 1,500–3000 W/m K, whereas, spherical particles (e.g. TiO_2_, Al_2_O_3_, MgO, ZnO etc.) possess thermal conductivities in the range of 1.4–48.44 W/m K.

As far as size of adding nanoparticles is concerned, it is also of great importance as it affects the effectiveness of the hybrid nanofluid. Different sizes lead to the changes in thermal conductivity, viscosity, stability, Brownian motion, volume ratio. Unlike shape, size is usually measured with the help of electron microscopes. It is reported to be in the range of few nanometres to 210 nm. The size is controlled during the synthesis procedures and it has been found to greatly affect the thermal conductivity of hybrid nanofluid (Shah et al., 2020).

## Environment and Economic Considerations

A putative method to regulate the environmental and economic effects of an invention is its life cycle valuation. In this part of the review article, we will discuss the environmental and economic contemplations of nanofluid-assisted collectors. A conventional solar collector was used in comparison with a nanofluid-based collector by Otanicar and Golden situated in Phoenix, Arizona (Sajid Hossain et al., 2015). They compared the maintenance and capital cost of both the collector systems and found that these values were higher ($120 and $20) with respect to the nanofluid and conventional collector. On the other hand, for the greater efficiency and yearly solar division of the nanofluid-assisted solar collector, the annual saving of fuel cost, for both natural gas and electricity was larger than that of the conventional solar collector. Furthermore, high cost of the nanofluid-based collector guarantied the longer payback duration, however, at the expiration of the aforementioned useful life, it processed the similar life cycle reserves as a conventional collector. The personified energy for the nanofluid-assisted collector is 9% lesser. In addition to that, according to environmental perspective, the development of the nanofluid-assisted solar collector leads to the lesser amount of CO_2_ emissions (34 kg), whereas, in its processing it protects 50 kg annually in comparison with the conventional solar collector. The amounts of added emissions including NO_x_ and SO_x_ are lesser, so the changes are not extensive. During the expected lifetime (15 years) of a nanofluid assisted solar collector, more than 740 kg of CO_2_ can be compensated compared with conventional solar collector. A solar water heating system based on nanofluid was designed by Khullar and Tyagi (Noguez, 2005). They regarded this system as a substitute for the fossil fuel-based heating systems. They revealed that the liquefied petroleum gas and electricity could be saved up to 206 kg/year and 1716 kWh/year, respectively, which harvests substantial economic paybacks. Additionally, the emission of CO_2_ can be reduced to 2.2 × 10^3^ kg annually. Conclusively, we can say that the nanofluid-based solar collectors can minimize the environmental issues, but the major concern for this system is their cost which may render their usefulness in various fields for the fulfilment of the energy needs.

## Factors Affecting the Performance of Nanofluid Based Solar Collectors

In spite of the increasing interest in the applications of nanofluids in solar systems, there are certain difficulties and defects that might be taken in consideration with special emphases to control and address them to be used more comprehensively in the field of solar energy systems. Herein, the greatest challenges and difficulties of using the nanofluids in solar energy systems are discussed as follows:

### Stability

The stability is the major area of concern for the nanofluids, particularly when the high concentration of the nanoparticles (NPs) is used. As the NPs have high surface tension and surface to volume ratios, therefore, their conglomerate over time is high which may govern the stability challenges (Devendiran and Amirtham, 2016). Hence, the low stability reduces the thermal conductivity with respect to time (Raj and Subudhi, 2018). A number of approaches have been examined and implemented to increase the homogeneity and stability of the nanofluids based systems. Nevertheless, addition of surfactants referred to the most cost-effective technique for the said problem (Ghadimi and Metselaar, 2013; Xuan et al., 2013; Xia et al., 2014).

### Complexity

The complex synthetic approaches for the nanoparticles are also one of the leading challenges for the fabrication of nanofluid-based solar systems. As discussed earlier, there are two approaches which are commonly used for the fabrication of nanofluids, namely one step and two step methods. In both approaches, NPs are produced that unite reactants in the form of reduction reactions or ion exchange that in turn disturbs the performance of nanofluids in solar collectors (Yu and Xie, 2012).

### High Cost

The complex synthetic procedures of NPs make the nanofluids expensive. This embodies the main defect of nanofluid-based systems which hinder their use in the field of engineering (Pantzali et al., 2009; Gómez-Villarejo et al., 2017). Therefore, the lowermost weight fraction of NPs, which retains comparatively great thermal conductivity, should be engaged for the purpose (Raja et al., 2016).

### High Viscosity

An undesired pumping power is required in case of nanofluid-based system because of the high viscosity of the fluids compared with the conventional systems (Kole and Dey, 2013). As a final point, the exergy efficiency and high energy enhancements in the experimental investigations show an encouraging future in the use of nanofluids. On the other hand, it requires the instituting standards for executing escalated studies including nanofluids so as to improve the commercialization of nanofluids applications in solar collectors.

Although the use of hybrid nanofluids has opened new horizons for practical applications, yet there are still some issues which need to be addressed. There is dire need of commencing in-depth researches experimentally and theoretically to know the effect of factors exploiting the performance of hybrid nanofluids. For instance, increase in viscosity leads to rise in pumping power, which is a great concern for hybrid nanofluids. Stability of the suspended particles is another worth noting issue. More shapes of the added particles need to be analyzed. The use of phase change materials in combination with NPs is also needed to further explored.

## Nanofluids Applications in Solar Energy

**FIGURE 4:**
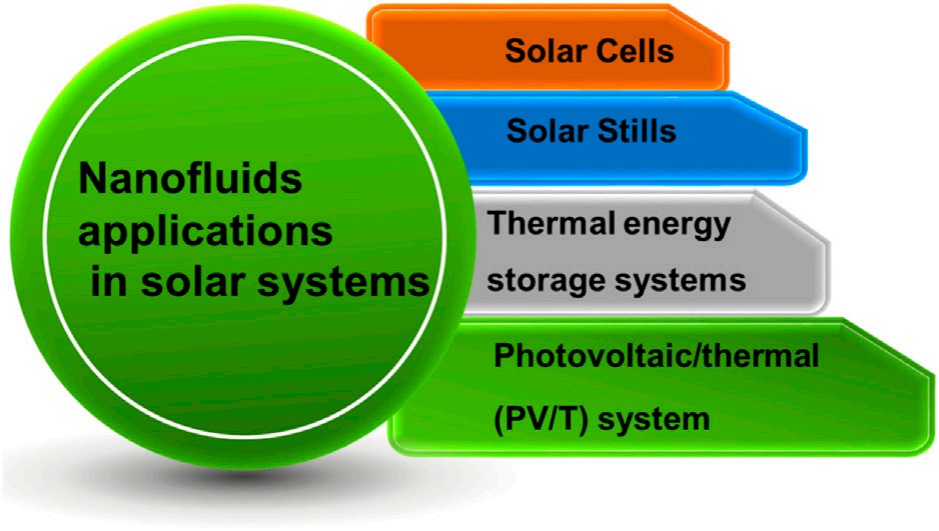
Schematic illustration of applications of hybrid nanofluids in solar energy systems.

A brief schematic illustration of the nanofluids’ application is portrayed in Figure 4.

### Solar Collector

**FIGURE 5:**
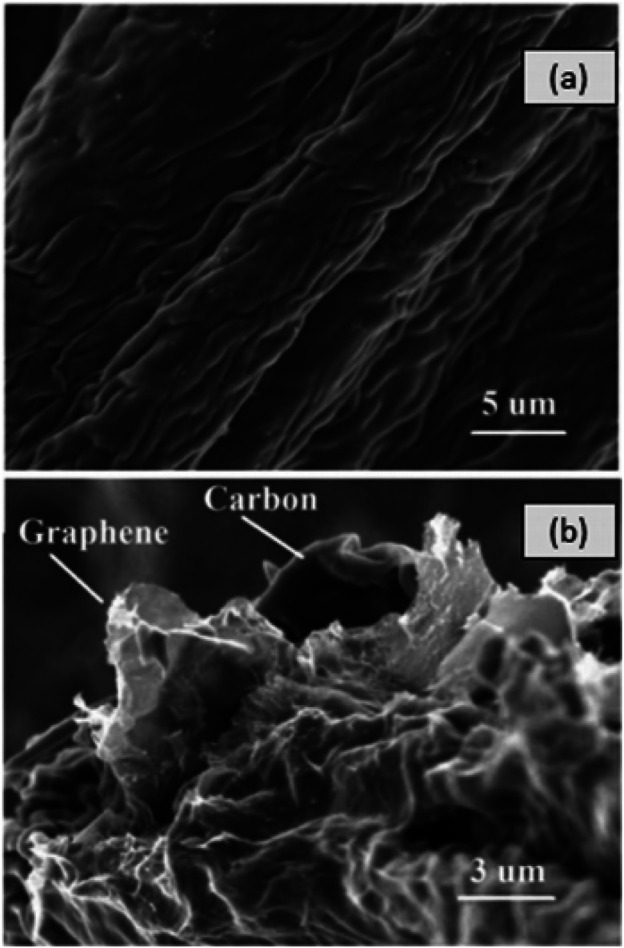
SEM images of **(A)** activated carbon (AC) and **(B)** activated carbon graphene (ACG) (Yarmand et al., 2016).

Solar radiations are converted into heat after getting absorbed on solar collectors. This generated heat can be used for various applications after conversion by the working fluid. Two major types of solar collectors include concentrating collectors and non-concentrating collectors (Kalogirou, 2004). Among these, non-concentrating solar collectors are specified for medium and low-temperature use. For example, space cooling and heating, desalination, and water heating, while concentrating solar collectors are used for elevated temperature applications e.g. electricity production. Nevertheless, these systems need intensive consideration regarding their low efficiency. In this regard, nanofluids have shown a great capability for improving the working efficiency of solar systems. In this section, a brief review has been made about the research overusing the nanofluids in solar collectors. The working of a direct absorption solar collector (DAC) (Tyagi et al., 2009) was examined briefly, which used aluminium water nanofluid as the adsorbent platform with glass surfaces on the top and entirely separated bottom. It was supposed to be a steady-state model with two dimensions for the transfer of heat. As the nanoparticle size increased, the collector efficiency also increased slowly. This happens because of the enhanced absorption coefficient that is directly influenced by the term D2 (particle size). The improvement of collector efficiency is certain because the volume fraction enhances greatly due to the lessening of sunlight passage *via* the collector. Results showed that with the same working parameters, efficiency for the direct absorption solar collectors with nanofluid was around 10% more as compared to the traditional models with pure water. Effects of different nanofluids e.g. graphite, silver, and CNTs have been studied in detail (Otanicar et al., 2010) by their direct absorption collector performance experiments. Later on, the results were compared with numerical models (Tiwari et al., 2013). Figure 5 explains the micro solar-thermal collector with a surface area of 3.5 cm^2^ and a channel depth of 150 μm. For the simulation of the solar spectrum, a super PAR64 lamp was used, while for the numerical model, previous work (Tyagi et al., 2009) was modified in which the radiative transport equation (RTE) coupled with the energy equation was used and emission terms were compared with the previous work.

Furthermore, the data was obtained from the experimental work of volume fraction vs. collector efficiency for various nanoparticles. The efficiency improved by increasing the concentration of particles, but after reaching the volume fraction up to 5%, a little bit of efficiency decreased. This decrease is because of water transmittance with a low concentration of particles which causes little heat. While for the nanofluids, high heat was expected with high particle concentration. We can summarize the efficiency increased because of three main reasons, changes in the optical properties of the fluid, decrease in the heat loss due to the dispersion of heat from the surface and lastly due to increase of the thermal conductivity. New model expectations say that there is a contrast about the effect of particle size results with Tyagi’s results (Taylor et al., 2011). In another study, a decrease in efficiency takes place due to the increase in the particle size. A theoretical study was made about the nanofluid-based flat plate collectors (Tiwari et al., 2013). They described briefly the increase of efficiency with 31% reduced potential in CO_2_ emission as compared to the typical models. It has been explored experimentally that the influence of the Al_2_O_3_-H_2_O nanofluid (Yousefi et al., 2012a) on the working of a solar collector by using ASHRAE standard. Results showed that a 28.3% increase in efficacy for 0.2 wt% nanoparticle concentrations as compared to the pure water. While another flat-plate solar collector with MWCNT-H_2_O nanofluids showed an increase of efficiency (Yousefi et al., 2012a). In another theoretical study, the use of nanofluid receivers in power tower solar collectors was studied (Bani-Hani et al., 2016), while at laboratory scale dish receiver, nanofluid were also used and an increase in the efficiency as compared to the base fluid was found. In another study (Sani et al., 2011), investigations were made about the potential of SWCNHs suspended in ethylene glycol, which was found as a better option for the exploitation in solar collectors. Another theoretical study (Khullar et al., 2012) was made about the increase in capacity of solar irradiance absorption for the NCPSC and experimental results were compared with traditional parabolic solar collectors. Notably, 5–10% of greater efficiency was observed than the conventional models. Heat transfer modeling was studied for different nanofluids (Kasaeian, 2012; Kasaeian et al., 2012) and also a numerical study about the increase in the heat transfer were reported for Al_2_O_3_/synthetic oil nanofluid in a parabolic trough collector tube (Abdelrahman et al., 1979). Surface absorbers are mainly used for solar energy transformation to electricity or heat. In these systems, there is a main imperfection which is the difference in temperature between absorber and heat transfer fluid. This difference occurs due to thermal resistance between interfaces. One solution to minimize heat loss is the volumetric absorption, in which a volume of heat transfer fluid absorbs the solar radiation directly. To attain the improved properties by adding small size solid particle in the base fluid was also studied (Khullar et al., 2012). Veeraragavan et al. (2012) revealed the concept of an analytical model for volumetric solar flow receivers by suspending NPs in the base fluid. The effect of different changes in nanofluids volumetric receivers were studied experimentally and theoretically. Theoretical studies supposed and proved a model with one-dimensional transient heat transfer and an improvement in the working efficiency of the receiver with increased nanofluid height (H) and intense solar flux was observed (Lenert and Wang, 2012). In experimental section of a study, carbon with cobalt NPs coatings was added to Therminols VP-1 in a volumetric receiver with a liquid base. Below a temperature of 700 K, an increase of nanofluid height decreased the efficiency of a receiver. While for the temperature range of 800–1200 K, efficiency increased, and no effect was noted for the temperature of more than 1300 K. Chances of volumetric absorption for the straight steam collection media were studied (Taylor et al., 2012a) in which laser light was used as a radiation source with a wavelength of 532 nm with three types of absorbing media including black painted surfaces, black dyes, and nanofluids. The temperature increased above 300°C for pure water but nanofluids got lower temperature although the generation of vapors increased up to 50%. It was assumed that the use of nanofluids increased the volumetric absorptions, so direct steam nanofluids collectors could improve the quality and conversion efficiency of the light into steam. In another study, Kandasamy and co-workers (Kandasamy et al., 2012) investigated about the Hiemenz flow of Cunanofluid on a porous wedge plate. It plays a vital role in the volumetric absorptions of the incident solar radiations and the transfer of the thermal energy to the fluid. Worth noting is that if the nanofluid is too thick then a thin layer will absorb the light resulting in the loss of thermal energy. Additionally, if nanoparticle concentration would be very small, a major part of the light will be transmitted, hence it is important to get a suitable amount of the nanoparticles for the volumetric absorption. It can be observed that the thermal resistance in a solar plant belongs to a surface-based and volumetric-based collector and it is clear that the thermal resistance is far less for a volumetric based collector. In a previously mentioned study (Otanicar et al., 2010) on a DAC, it was noted that volumetric absorption caused the highest temperature to rise around the central part as compared to the surface and minimum loss of heat took place. This is an imperative process in the volumetric receivers, which causes an increase in efficiency. Optical attributes of the base fluid can be changed by the NPs and they can act as the optical filters for different applications (Taylor et al., 2012b). Researchers studied the Ni nanoparticle suspensions and explored their radiation absorption features (Kameya and Hanamura, 2011). Results revealed that the absorption enhanced greatly for the wavelength in visible and near-infrared regions, while the coefficient of absorption remained constant for the infrared region. It was supposed to be a useful mechanism for DAC. Optical properties investigations (Han et al., 2011) showed the rheological behaviors and carbon black-water nanofluids’ thermal conductivity for solar absorption. Results showed the great potential of carbon black nanofluids being used up for solar systems. Studies about the effect of aluminium-water nanofluids in the direct solar collectors were also made (Saidur et al., 2012). Aluminium nanoparticles increased the absorption of light in visible light with shorter wavelengths irrespective of the low extinction coefficient. According to the authors, particle size had the least impact on the optical features, but for the sake of benefit using Rayleigh distribution, the size of the particles should be lower than 20 nm. After getting the optimum optical characteristics, the volume fraction should not be increased for the prevention of stability and agglomeration of the suspension. For the direct absorption solar collectors, researchers found the optical properties of the different nanofluids (Taylor et al., 2011), in which nanofluids stability was a noteworthy aspect for their performance and supplied by the addition of pH buffers, surfactants, or the chemical treatments. The effect of pH changes on the thermal conductivity of the nanofluids has been studied by many researchers (Mingzheng et al., 2012; Younes et al., 2012). In one study, the effects of pH changes for MWCNT-H_2_O nanofluid on the performance of a flat plate solar collector were observed in detail (Saidur et al., 2012). For the dispersion of the nanoparticles, a water-based MWCNT with 0.2 wt% was used in the presence of Triton X-100.

**FIGURE 6:**
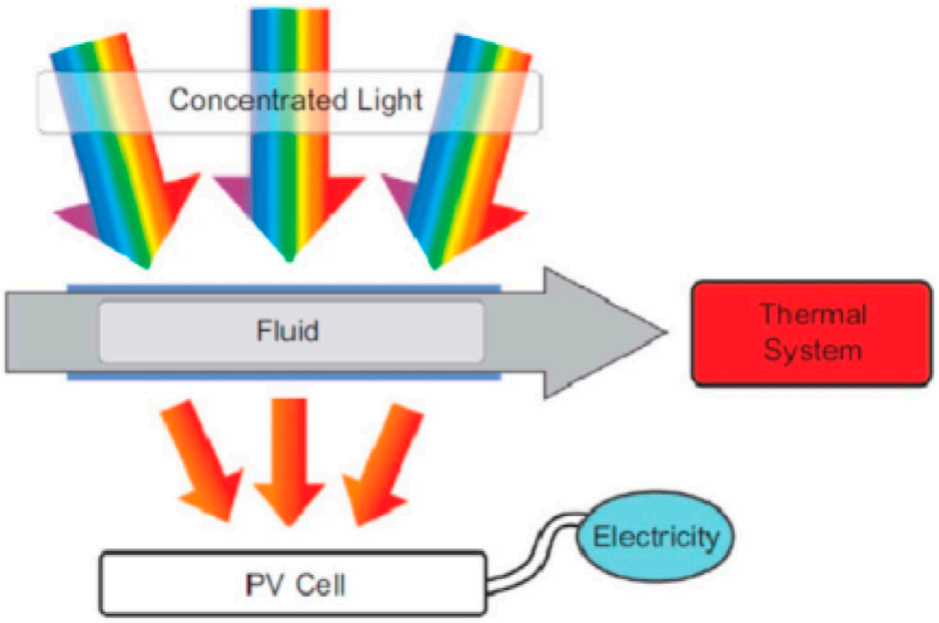
Sketch of the de-coupled PV/T system concept (Taylor et al., 2012b).

Figure 6 shows schematic of the experiment in which the efficiency of the collector has been illustrated. The efficiency of the system can be enhanced by nanofluids with control of sedimentation of the solid phase. Hence, for the use of nanofluids, there must be no sedimentation for the solid phase. For this purpose, sedimentation of solar plate collectors was studied, and a useful solution was analyzed to avoid this issue (Colangelo et al., 2013). The stability of various types of water-Al_2_O_3_ nanofluids with maximum stability of suspension around 1, 2, and 3 vol% was studied. An optical study was made for the heat transfer coefficient, h, thermal conductivity, k, and the sedimentation in a flat plate solar thermal collector. In this hot wire technique analysis, an increase in thermal conductivity had a direct relationship with the volume fraction reaching 6.7%–3 vol% of Al_2_O_3_. The convective heat transfer coefficient was also increased for both the turbulence and the laminar flow areas with 25% for water-Al_2_O_3_ nanofluids with 3 vol%. Moreover, it was claimed by the authors that the deposited solid phase was directly proportional to the average of the fluid velocity present inside the top and bottom areas. For the reduction of sedimentation in flat plate solar thermal collectors, various cross-sectional areas were used, and velocity was kept constant in the bottom header and top header parts. Additionally, it has been reported that the use of hybrid nanofluid can lead to enhanced thermal efficiencies in flat plate systems in comparison with single nanoparticles use and traditional fluids (Farajzadeh et al., 2018). The various types of hybrid nanofluids based on nanoparticles are illustrated in Table 2.

**TABLE 2:** Summary of nanofluids used in solar collector.

Nanofluid	Nanoparticle size	Result	References
Nitrate eutectic salts seeded with silica	10,20 and 30 nm	The 20 nm NP displayed a maximum enhancement in the average specific heat capacity (by ∼26.7%)	Hu et al. (2019)
Al_2_O_3_	20, 50 and 100 nm	The solar collector with 1.0 vol% Al_2_O_3_ nanofluid of 20 nm-NP and a mass flow rate of 0.047 kg/s showed the highest efficiency, 24.1% higher than that of the solar collector with water	Kim et al. (2017)
CeO_2_	25 nm	Experiments indicate that the highest rise in efficiency of the collector at zero value of is 10.74%, for volume fraction 0.066%, and for mass flux rate of 0.019 kg/s m^2^ compared to water	Sharafeldin and Gróf (2018)
CuO	40 nm	These CuO nanofluid with mass flow rate of 1 kg/min increases the collector efficiency about 21.8%	Moghadam et al. (2014)
f-GNP	20 nm	The highest thermal performance of a solar collector has reached 78% at mass concentration 0.1 mass% and flow rate 0.0260 kg s^−1^ m^−2^ which is 18.2% higher than water at the same flow rate conditions	Akram et al. (2019)
WO_3_	90 nm	The maximum enhancement in efficiency of the collector at zero value of was 13.48% for volume fraction of 0.0666% and mass flux rate of 0.0195 kg/s m^2^	Sharafeldin et al. (2017)
Al_2_O_3_	20 nm	These nanofluid incorporated increases the collector efficiency about 23.6%	Mirzaei et al. (2018)
SiO_2_	20–30 nm	Solar collector performance is improved up to 0.92 with SiO_2_ nanofluid	Jouybari et al. (2017)
(Mixture of Al_2_O_3_ and TiO_2_)	20 and 15 nm	Increasing the concentration of the 7 nanofluid mixture from 0.1 wt% to 0.2 wt% will result in approximately 5% 8 improvement in the thermal efficiency of the solar collector	Farajzadeh et al. (2018)
MgO	40 nm	Experimental observation establishes thermal efficiency enhancement 9.34% for 0.75% particle volume concentration at flow rate 1.5 lpm	Verma et al. (2016)
GNPs	<100	The results indicate that dispersing Graphene in the base fluid can increase thermal efficiency of the solar collector up to 18.87%	Ahmadi et al. (2016)
CF-MWCNTs + CF-GNPs + h-BN	Varied between 236.1 and 456.3 nm	The improvement in the thermal efficiency was up to 85% using hybrid nanofluid	Hussein et al. (2020)
CuO + MgO + MWCNTS	—	The performance of MgO hybrid was superior to that of CuO hybrid and close to the MWCNTs hybrid	Verma et al. (2018)
Al_2_O_3_+CuO	CuO = 29 nm; Al_2_O_3_ = 40 nm	The improvement in the solar collector’ efficiency was found to be 45%	Tahat and Benim (2017)

### Solar Stills

For the freshwater production, the solar distillation system as an alternative solution has received high attention. In the literature, there are still many solar designs that have been proposed e.g., active and passive solar still. Primarily, this production of solar still is dependent on the process of heat transfer and its working temperature. By improving the thermal properties, the heat transfer coefficient can be enhanced by NPs inclusion into the base fluid water (Sahota and Tiwari, 2016a; Sharshir et al., 2017).

The addition of nanoparticles (such as MnO_2_) to the solar stills increases the rate of production of distilled water (Prasad et al., 2011). It was noted that by adding 100 g of MnO_2_ per 6 L of liquid could be a suitable amount for graphite-like solids to get the maximum efficiency. The size of the MnO_2_ particles was found to be 0.5 mm. It has been studied that the use of nanofluids with glass cover cooling causes an increase in solar still’s working efficiency. Their results showed improved solar still productivity up to 44.91 and 53.95% while using the copper oxide micro-flakes of graphite. Moreover, copper oxide and graphite particle usage increased the output yield around 47.80 and 57.60%. For the three various nanofluids, an expression of the specific equation of passive DSSS was developed (Akash et al., 1998) in which an analysis was made for the optimal amounts (0.25%) of metal NPs. Greater thermal efficiency was found for the nanofluids (Al_2_O_3_ 50.34%; CuO 43.81%, and TiO_2_ 46.10%) in comparison with the pure fluid (37.78%). Experimental studies were made to observe the effect of using various absorbing materials in a solar still and the results revealed that water productivity on daily basis was improved up to 45 and 38% by using black ink and black rubber mat as absorbers, respectively. In another study, Gnanadason and co-workers (Gnanadason et al., 2012) assessed the effect of using nanofluids in a vacuum single basin solar still. The addition of CNTs into the water basin showed efficiency improvement of solar stills up to 50%. An increase of the productivity yield of the passive double slope solar still was found by using three different amounts (0.04, 0.08, and 0.12%) of the Al_2_O_3_ NPs in the water of base fluid. It showed that Al_2_O_3_ nanoparticles with 0.12% amount yield 12.2 and 8.4% increase for the 35 and 80 kg pure fluid, respectively, in comparison with the pure fluid (Sahota and Tiwari, 2016b). The performance of a single basin solar slope still was analyzed experimentally (Elango et al., 2015) with and without using the nanofluids. Results showed that the solar still with the nanofluid Al_2_O_3_ produced 29.95% more as compared to the pure water. In another study, single basin solar still with different absorbents such as charcoal, violet dyes, and dissolved salts to enhance the water absorptivity for the solar radiations was analyzed (Nijmeh et al., 2005). The salts used were named as potassium dichromate (K_2_Cr_2_O_7_) and potassium permanganate (KMnO_4_) and they improved the efficacy and productivity of the solar stills. Interestingly, potassium permanganate enhanced the efficiency up to 26%. In a recent research, porous materials and nano-medium played an important role inside the absorber and enhanced the distillate outcome up to 38 and 92% for the light and dark sponges, respectively, which is far better than the pure fluid. Rabbi et al. reported the improvement in performance of the solar still by employing hybrid nanofluid consisting of Al_2_O_3_-SiO_2_-water. Interestingly, exergy and thermal efficiency were noted to be 0.82 and 37.76% respectively at 4.99 kg m^−2^ day^−1^. The better performance was ascribed to the collective effort of inside temperature of heat exchanger, flowrate of nanofluid, and nanoparticles’ concentration (Rabbi and Sahin, 2021).

### Thermal Energy Storage Systems

The efficiency of solar thermal systems majorly depends on the specific heat of the fluid and temperature of the thermal energy storage systems (TES) e.g., fatty acids and mineral oils (Kearney et al., 2002). Later on, molten salt was used in the CSP systems due to the storage of more energy as compared to the synthetic oil and this is related to the reason of its stability at elevated temperature e.g. 600°C (Janz et al., 1979; Shin and Banerjee, 2010). In solar plants, the performance of the power plants increased from 54% at 400°C to 63% at 560°C. Furthermore, the use of molten salt is less expensive, environment friendly, and safer as compared to traditional heat transfer fluid. But there is a major problem regarding the use of molten salt, it has a high freezing point and extremely bad thermophysical characteristics which cause an increase of TES’s requirements. For nonstop operations of thermal systems, it’s difficult to use dispersed salts with nanoparticles in TES with high temperature nanofluids. The elevated specific heat capacity of nanofluids can decrease the required amount of thermal energy, which results in a decrease in the size as well. Numerous studies showed that the specific heat capacity of the environmental nanofluids reduced with an enhancement of the NPs volume fractions, while nanofluids with molten salt showed the opposite results related to the upgrading of specific heat capacity (Akash et al., 1998; Prasad et al., 2011).

There was an abnormal increase in the specific heat capacity and elevated temperature of nanofluid was observed (Shin and Banerjee, 2011b). Precisely, 1% mass concentration of the silica NPs was used and the results revealed that appropriate heat capacity was improved up to 14.5%. Moreover, Ercole et al. (2017) evaluated the thermal efficiency of the eutectic molten salt-based nanofluids. Results of specific heat of solar nano-salts with 1% (by weight) amount of NPs showed good thermal capacity as compared to the other amounts. There were four different sizes of silicon dioxide NPs in molten salt i.e., in potassium carbonate and lithium carbonate to get the elevated temperature operating fluids (Tiznobaik and Shin, 2013). Results showed an improvement of 25% in the specific heat capacity of the nanofluids by using nanoparticles with 1% of the mass concentration without considering the diameter of the nanoparticles. Suitable heat capacity of the molten salt-based alumina nanofluids decreased with an extra NPs addition and it is in agreement with previously done studies on the conventional nanofluids.

### Photovoltaic/Thermal System

Conversion of sunlight into electrical and thermal energies by using photovoltaic/thermal (PV/T) devices has been reported recently (Lu and Huang, 2013; Yang and Athienitis, 2016; Abdelhamid et al., 2016; Guo et al., 2017). This is possible by using a solar cell, and it transforms the sunlight into electricity by using semiconductors. This technology is being used for the conventional PV cells and has a prominent effect on the efficiency of photovoltaic cells. It also overcomes the major drawbacks of the PV cell’s incapacity to absorb the sunlight from the whole spectrum (Chu and Meisen, 2011; Mohammad Bagher et al., 2015; Green and Bremner, 2017). So, the integration of the thermal with solar cells utilizes a major portion of solar energy, which improves the overall thermal and electrical efficiencies. Many experimental and theoretical studies were made on the electrical and thermal efficiency of the PV/T systems (Rosell et al., 2005; Ji et al., 2009; Al-Waeli et al., 2016). To improve the efficiency of PV/T by one of the most efficient methods is to apply the nanofluid as a coolant in PV/T systems to facilitate the process of heat transfer (Saroha et al., 2015). Experimental investigations were made on the possibility of the use of SiO_2_/water nanofluid in a PV/T system. After that, evaluations were done for the electrical and thermal efficiencies by using the thermodynamics laws, and the out-coming electrical energy was converted into the corresponding thermal energy. The thermal efficiency by using nanofluids with 1 wt% and 3 wt% was increased up to 7.6 and 12.8% than pure water. Furthermore, the integration of thermal collectors with a PV system led to an increase of total energy by 19.36% (pure water), 22.61% (1 wt% nanofluid), and 24.31% (3 wt% nanofluid). Effects of environmental parameters were also studied, for instance, absorbed solar energy along with the temperature of the inside working fluid were analyzed using Al_2_O_3_/water nanofluid and pure water. It was observed that the increase in the incident solar radiation caused a reduction in electrical efficiency, but the thermal efficiency was not affected after the first rise. Furthermore, the rise of the inlet fluid temperature led to decreased electrical performance, and the thermal efficiency did not show any noticeable change (Khanjari et al., 2017). Theoretically, the effects of using alumina/water and Ag/water nanofluids on the efficiency of the PV/T system were also investigated (Khanjari et al., 2016). Results revealed that the thermal efficiency along with the heat transfer coefficient were enhanced by the increment of the volume fractions of the NPs, moreover, an increased utilization of the photovoltaic hybrid system was reported because of its lower cost to generate solar electricity with high performance (Chow, 2010). Solar cells directly convert incident solar radiation into the electricity in a hybrid system, while the leftover absorbed solar energy is converted to the heat by circulating nanofluids. Later, a heat exchanger is applied to convert the thermal energy into air or water, which can be further used in desalination, space and building heating and air ventilation applications (Ibrahim et al., 2011). The optimum efficiency can be obtained with a mode system efficiency of 20% by a typical 200-sun concentration. Furthermore, a reduction in the temperature of the inside fluid and an increase in the fractional volume of the nanoparticles can improve the system’s efficiency. A new design of the PV/T system of the dual concentration was proposed in further studies (Xu and Kleinstreuer, 2014b), in which both electrical and thermal energy with remarkably improved efficiencies and reduced solar cell material were observed. Dilute nanoparticles suspended in the fluid were used for the first time for the improvement of PV/T system’s efficiency. Results illustrated that the use of a controlled flow rate resulted in nanofluid temperature of 62°C at outlet, and the efficiency of the system as a whole enhanced up to 70% with the contribution of the electrical (11%) and thermal (59%) efficiencies. The consumption of nanofluid-based optical filters for the hybrid PV/T applications has also been reported which gave superior solar-weighted efficiency to the base fluids and also provided better efficiency as compared to the traditional optical filters in the range of ultraviolet or infrared wavelengths of the solar radiation. Furthermore, researchers studied different types of PV cells such as CdTe, InGaP, InGaAs, Ge, and Si. Different types of the NPs were used based on the types of the PV cells, for example, an ideal filter was specifically used for Na-Si PV cell that must have a material response curve and absorb the wavelength greater than 1.125 mm and less than 0.75 mm. The outcomes showed that most of the nanoparticles with a volume fraction of 0.0011% were needed to obtain the optical performance of a PV/T system. For the first time (Hjerrild et al., 2016), researchers assessed the nanofluid-based optical filters for the hybrid PV/T applications in which two nanofluids with water base were used by suspending MWCNTs and Ag–SiO_2_ nano-discs. Around 30% of thermal and electrical efficiency was increased by using Ag–SiO_2_ nanofluids than that of base fluid optical filter. Experimental studies were conducted on the use of a concentrating PV/T collector with an optical filter of the nanofluid base. Cu_9_S_5_ nanoparticles of the oleylamine solution were used in the optical filter. Applying this nanofluid optical filter increased the performance of the PV/T collector and it was 17.9% greater than without using an optical filter (An et al., 2016). Exegetics and environmental studies (Hassani et al., 2016) were made for the evaluation of the exergy and environmental influence of nanofluid use based on carbon nanotube/water nanofluid optical filter in the PV/T hybrid system, and then its comparison was made with the performance of a traditional PV and PV/T systems. These analyses showed that the configuration of PV/T were produced around 1.3 MWh/m^2^ of exergy per year and decreased the PV/T system emission around 448 kg CO_2_ eq. m^−2^ yr^−1^. Results of PV/T systems with two different channels showed that more use of the nanofluid volume fraction increased the electrical efficiency specifically for the GaAs and Si PV cells by 5.8 and 4.5%, respectively. Nanofluids as a cooling agent in the PV/T systems to increase the transmission of the heat process has been examined by different researches. But the influences of some types of the nanofluids e.g., CuO/water showed a remarkable increase in the SCs performance, and were not studied before. Moreover, the use of hybrid nanofluids in the PV/T systems should be studied. In this regard, efforts were made to use hybrid nanofluids as optical filter and coolant in PV/T systems. The results showed that thermal efficiency was influenced by inlet velocity of fluid. The maximum rise in thermal and electrical efficiencies was found to be 5.4 and 2.14% for hybrid nanofluid. Pressure drop was also found to be reasonable (214.78 Pa) in case of hybrid fluid (Karaaslan and Menlik, 2021). There is a need of economic studies for the assessment of the filter’s performance with its manufacturing cost, working cost, and the amount of produced energy as a result. Nanofluid-based optical filter increases the whole efficiency of the PV/T system as compared to the pure fluids due to highest solar radiation absorption. Hence, the use of the plasmonic nanofluids in the PV/T systems must be further studied, keeping in view thermal and economic considerations.

## Challenges

Nanostructures with size less than 100 nm have been widely applied in the industrial exploitations, such as medical imaging, drug delivery, gene therapy, and manufacturing of new products, but the dreadful consequences of these nanoparticles on human health are yet to be identified. This is because of the unidentified size, shape, and chemical compositions of the different nanoparticles. Furthermore, the common nanoparticles have high surface areas as compared to the other bulk materials, so they can damage the human body and the ecosystem more as compared to the bulk particles. It has been reported that superfine TiO_2_ particles were present in all the sections of lung tissues and cells (Geiser et al., 2005). Additionally, particle addition was made *in vitro* cells through different interactions such as diffusion or adhesions and direct admittance to the organelles, DNA, and intracellular proteins. Strongest superconductive nanoparticles are CuO, which make them appropriate for several applications e.g. electronics, solar systems, batteries, and many others (Bondarenko et al., 2013). In 2014, more than 300 tons of CuO NPs were produced in the US (Mashock et al., 2016). Copper materials are the biggest reason behind the environmental pollution, such that Cu ions and CuO NPs contain great toxicity responses for marine entities (Schrand et al., 2010) and earthworms (Unrine et al., 2010). CuO nanoparticles discharge the copper ions into the solutions which create the toxic effects. Toxicity of the copper oxides and their effects on the human body and environment were studied briefly (Mashock et al., 2016). Moreover, the effects of CuO NPs and copper sulphate on the cells and neurons were studied. People after exposure to the CuO NPs witnessed the degenerating dopaminergic neurons up to 10%. Results also revealed that CuO NPs had a difficult toxic nature. A review about ecotoxicity (Bondarenko et al., 2013) revealed the toxicity of the CuO, silver, and ZnO NPs and they could fight (such as nanosilver) against the growth of fungi, bacteria, and algae. Their discharge may be harmful to non-target organisms as well. High-cost production is another noticeable challenge for the different nanofluids’ applications. Moreover, agglomeration and instability of the nanoparticles, pumping power, and the pressure drop are other issues. For the sake of application, nanoparticles must be highly pure with low defects. The production cost of the nanoparticles is always high due to achieving the exact properties by using the most reliable and precise processing. It has been studied that heat exchangers cause the high cost of nanoparticles (Lee and Mudawar, 2007). The viscosity of the fluids increases due to nanoparticles’ addition; hence, the pressure goes down, which causes the increase in pump power. Pumping power was measured experimentally for nanofluids flowing in a system as well as in elbows, expansions, and straight tubing (Routbort et al., 2011). The addition of nanoparticles increases the pumping power in contrast to the base fluid. Agglomeration and instability of the nanoparticles is the big problem at elevated temperature, specifically under natural circulations. Hence, for the high-temperature applications, there should be given more attention to the selection of the nanoparticles.

## Conclusion and Future Outlook

Conclusively, the benefits and applications of hybrid nanofluid in solar energy and photovoltaic/thermal (PV/T) system are discussed. The studies presented in this review article reveal that the use of hybrid nanofluids in solar energy and photovoltaic/thermal (PV/T) systems have promising impacts on the energy efficiencies of the devices. We can summarize all the findings as follows: 1) In certain cases, the efficiency of these solar systems can be enhanced up to 60%. This enhancement can be ascribed to the increased thermal conductivity of the system which in turn results in the increased value of Nusselt number in case of hybrid nanofluids as compared to the pure fluids. 2) Furthermore, the introduction of hybrid nanofluids results in the improved heat transfer accompanied with increase in photo conversion efficiency and enrichment of optical properties of photovoltaic/thermal (PV/T) system. 3) Concentration and type of hybrid nanofluid are influential parameters for the increased performance of the solar energy and photovoltaic/thermal (PV/T) systems. 4) In addition to the properties of hybrid nanofluid, the operating modes such as inlet temperature and flow rate of the solar collector are also the additional factors for the enhancement of efficiency. Finally, it can be concluded that focusing on the kinds of nanostructures and base fluids is a useful idea to discover more suitable hybrid nanofluids based on optical and thermal properties.
